# Crosstalk between autophagy and apoptosis in initiating antitumor immune responses in human lymphoma cells

**DOI:** 10.37349/ei.2025.1003216

**Published:** 2025-09-11

**Authors:** Kayce Blumenstock, Faisal F. Y. Radwan, Vandana Zaman, Narendra L. Banik, Azizul Haque

**Affiliations:** 1 Department of Pharmacology and Immunology, Medical University of South Carolina, Charleston, SC 29425, USA; 2 Ralph H. Johnson Veterans Administration Medical Center, Charleston, SC 29401, USA; 3 Hollings Cancer Center, Medical University of South Carolina, Charleston, SC 29425, USA; 4 Department of Neurosurgery, Medical University of South Carolina, Charleston, SC 29425, USA

**Keywords:** triterpenoid, autophagy, apoptosis, crosstalk, HLA class II, CD4^+^ T cells, immune recognition

## Abstract

**Aim:**

Despite advances in lymphoma treatment, resistance to conventional therapies and insufficient immune-mediated tumor clearance remain major challenges. This study investigates the dual antitumor mechanisms of the mushroom-derived triterpenoid, ganoderic acid DM (GA-DM), exploring its ability to induce programmed cell death while enhancing immune recognition in diffuse large B-cell lymphoma (DLBCL).

**Methods:**

DLBCL cells (DB and Toledo) were treated with GA-DM (0–40 μM), and cell viability was assessed via MTS assay. Apoptosis was evaluated through caspase-3 activation and inhibition by ZVAD-FMK, while autophagy was measured via LC3 protein expression. Flow cytometry analyzed HLA class II surface expression and antigen presentation to CD4^+^ T cells (via IL-2 production), with autophagy’s role further confirmed using the inhibitor 3-MA.

**Results:**

GA-DM exhibited potent and dose-dependent cytotoxicity against DLBCL cells, with concentrations of 30–40 μM inducing over 60% cell death within 24 h. Mechanistic studies revealed that GA-DM activated the intrinsic apoptotic pathway, as evidenced by caspase-3 cleavage and the significant reduction in cell death upon ZVAD-FMK treatment. Concurrently, GA-DM treatment upregulated the autophagy marker LC3-II, indicating the induction of autophagy. Strikingly, GA-DM also enhanced the immunogenicity of lymphoma cells by increasing surface expression of HLA class II molecules. This led to improved antigen presentation and subsequent activation of CD4^+^ T cells, as demonstrated by a 2.5-fold increase in IL-2 production (amount of IL-2 in pg/mL) compared to untreated controls. The critical role of autophagy in this process was confirmed by the near-complete abrogation of HLA class II-mediated T-cell activation upon 3-MA treatment.

**Conclusions:**

GA-DM synergistically induces apoptosis and autophagy while promoting immune-mediated tumor clearance through enhanced HLA class II antigen presentation. These findings highlight GA-DM as a promising multi-modal therapeutic candidate for lymphoma immunotherapy.

## Introduction

B-cell lymphoma is a type of cancer that originates in the B-cells, a type of white blood cell involved in the immune system [1, 2]. These B-cells are responsible for producing antibodies and presenting antigens to T cells that help the body fight off infections. When they become cancerous, they can expand uncontrollably and form a tumor and spread through the lymphatic system. There are two main types of B-cell lymphomas: (a) non-Hodgkin’s lymphoma (NHL) and (b) Hodgkin’s lymphoma [3]. NHL is the most common hematologic malignancy, capable of invading the brain, meninges, and nerve roots, leading to high lethality. Diffuse large B-cell lymphoma (DLBCL) is the most common form of aggressive NHLs [4, 5], accounting for nearly 40% of all lymphomas. With multiple agent chemotherapy, the overall survival is approximately 50% at 5 years, including those who are completely cured or have relapsed disease.

Treatment for B-cell lymphoma can vary depending on the specific type, stage, and many other factors. Common treatments include chemotherapy, radiation therapy, immunotherapy (e.g., monoclonal antibodies like rituximab, checkpoint blockers, etc.), and stem cell or bone marrow transplants [6–8]. Overall, the prognosis for B-cell lymphoma varies depending on a variety of factors. Some forms are very treatable and can easily go into remission, while others can be more aggressive and require more intensive treatment. Most patients with aggressive NHL undergo therapy with R-CHOP (R = Rituximab, C = Cyclophosphamide, H = Doxorubicin Hydrochloride/Hydroxy-daunomycin, O = Vincristine Sulfate/Oncovin, P = Prednisone) or a similar regimen. In aggressive DLBCL, approximately two-thirds of patients attain a cure with commonly used R-CHOP [9]. While the prognosis for many people with B-cell lymphoma has improved significantly due to advances in different treatments, there are still challenges in terms of disease resistance, drug side effects, treatment accessibility, and long-term outcomes. Investigators continue to explore new therapies, including targeted chemotherapies and immunotherapies, such as chimeric antigen receptor-T cell (CAR-T) therapy [10], to overcome some of these limitations and improve the overall prognosis for B-cell lymphoma patients.

Cell death is a natural process essential for maintaining health, involving both programmed and accidental cell death mechanisms. Apoptosis is a form of programmed cell death, and its regulatory pathways are important in cancer, including B-cell lymphomas [11–14]. In a healthy immune system, apoptosis helps remove damaged or infected cells, although the balance between pro-apoptotic and anti-apoptotic signals in lymphomas is often disrupted. This can help lymphoma cells evade normal cell death processes, contributing to the uncontrolled growth of malignant cells. By investigating the role of apoptosis in lymphoma, researchers are developing novel therapies to selectively target and induce cell death in malignant cells while sparing normal healthy cells. Autophagy may help lymphoma cells survive under stress conditions like nutrient deprivation or chemotherapy treatment [15, 16]. Malignant cells often increase autophagy to cope with cellular metabolic stress, as it allows them to recycle their own cellular components to maintain energy and prevent cell death. Studies have also shown that lymphoma cells can become more resistant to chemotherapy if the autophagy process is increased [16]. This happens because the autophagy process helps them avoid apoptotic cell death, allowing the malignant cells to continue proliferating even in the presence of chemotherapeutic drugs. By modulating their immune components, lymphoma cells could avoid being targeted and eliminated by cells such as T cells.

A recent study suggests the dual-nature of autophagy in lymphoma may make it a challenging target for designing therapy [17]. Investigators are exploring ways to modulate autophagy in treating aggressive B-lymphoma, using agents to block survival or promote activators to trigger cell death, depending on the circumstances. While autophagy can support tumor cell survival and growth, it can also lead to cell death under favorable conditions [14, 18, 19]. The interplay between autophagy and apoptosis is a complex and highly regulated process that may play an important role in maintaining cellular homeostasis and determining cell fate and disease. Both autophagy and apoptosis are important mechanisms for determining cell survival and death [20–22], and their crosstalk is crucial in guiding cancer cell survival and death. Autophagy is also a critical player in immune regulation, altering inflammation, antigen presentation, and immune cell homeostasis. Its interplay with the immune system is complex and dependent on multiple factors, making it a promising target for therapeutic interventions in inflammatory and immune-mediated diseases.

Antigen presentation is a critical regulatory process where antigen-presenting cells (APCs) capture antigens, process and present them on their surface for recognition by T cells. Antigen presentation and autophagy are two important and interconnected processes in the immune system that help the host recognize and respond to infectious agents, as well as maintain cellular homeostasis [23, 24]. They also play crucial roles in immune surveillance, clearance of infectious agents, and maintaining cellular homeostasis, health, and disease. Their interplay ensures induction of optimum immune responses and helps prevent the growth of malignant cells. Understanding the interplay between apoptosis and antigen presentation is critical for developing therapies for infections, autoimmunity, and malignant diseases.

The majority of human B-cell lymphomas express HLA class II proteins, which can bind antigenic peptides, and present HLA class II-peptide complexes to CD4^+^ T cells for their immune recognition [25–27]. Tumor-derived antigenic peptides can readily get access to vacuolar compartments by sequestration in double-membrane organelles, which can then fuse with vesicles of the endolysosomal compartments, where peptides can be loaded onto HLA class II proteins [28, 29]. Considering the fact that immune escape can contribute to the malignant growth of cells and standard chemotherapies induce treatment-associated toxicities, it is crucial to search for natural products that are less cytotoxic and can kill the cells or stimulate the immune response. One attractive natural product could be ganoderic acid DM (GA-DM), a triterpenoid extract of *Ganoderma lucidum* [30]. The current study aims to investigate whether GA-DM can influence B-lymphoma growth and presentation of immunogenic peptides to CD4^+^ T cells via HLA class II molecules. We show in this study that GA-DM treatment influences autophagic and apoptotic events as well as immune activation in human B-lymphoma cells, which may be important in determining tumor growth and anticancer therapy.

## Materials and methods

### Cell lines

Human DLBCL cell lines, DB [American Type Culture Collection (ATCC) Cat #CRL-2289] and Toledo (ATCC Cat #CRL-2631), were cultured in complete RPMI-1640 (Cat #10–040-CV Corning, Manassas, VA) medium supplemented with 10% fetal bovine serum (FBS) (Cat #SH30088.03 HyClone, Logan, UT), 50 IU/mL penicillin, 50 μg/mL streptomycin (Cat #30–001-CI Corning, Manassas, VA), and 1% L-glutamine (Mediatech) at 37°C in 5% CO_2_ [31–33]. DB and Toledo cells were obtained from the ATCC. Authentication of cells is performed by short tandem repeat (STR) analysis in the Molecular Biology facility at MUSC. Cell lines are also monitored for mycoplasma contamination every six months using the MycoAlert Mycoplasma Detection Kit (Lonza). DB and Toledo cells were transduced using retroviral vectors for constitutive expression of HLA-DR4 proteins (DRB1–0401) with linked drug selection markers for hygromycin and histidinol (Sigma-Aldrich, St. Louis, MO) resistance [32, 33]. Expression of cell surface HLA-DR4 proteins was confirmed by flow cytometric analysis using the DR4-specific mAb, 359-F10 [34, 35]. The T cell hybridoma line 2.18a was cultured in complete RPMI-1640 with 10% FBS, 50 IU/mL penicillin, 50 μg/mL streptomycin, and supplemented with 1% L-glutamine (Mediatech) and 50 μM β-mercaptoethanol (β-ME) (Sigma-Aldrich, Cat #M6250–10ML) as described previously [32, 36]. T cell hybridoma lines were generated many years ago and tested in our lab in multiple experiments [32, 34]. These hybridoma lines were stored in liquid nitrogen and used in successive experiments.

### MTS cell viability assay

GA-DM, originally isolated from the *Ganoderma lucidum* mushroom, was purchased from a vendor named Planta Analytica, LLC (Danbury, CT) (Cat #G-032) [37]. The purity of GA-DM was tested by the vendor as 99.9% using LC/MS analysis. GA-DM powder was dissolved in dimethyl sulfoxide (DMSO) (Cat #D2650 Sigma, Burlington, MA) to make a 10 mM stock solution for use in the cytotoxicity assay. For all kinds of GA-DM treatments, the DMSO final concentration used was ≤ 0.1%. Human DLBCL lines DB and Toledo (2 × 10^5^ cells/well in 100 μL culture medium) were incubated with GA-DM (final concentrations 0, 10, 20, 30, and 40 μM) for 24 h at 37°C in 5% CO_2_ in a 96-well plate (Corning). DB cells were also treated with 30 μM of GA-DM for 24 h in the presence or absence of a pan-caspase inhibitor (Z-VAD-FMK, 50 μM) (R&D Systems #FMK001, Minneapolis, MN) overnight [37, 38]. Following treatment, cell viability was quantitated using the CellTiter 96 Aqueous One Solution Cell Proliferation Assay (Cat #G3581 Promega, Madison, WI) [38]. MTS (20 μL) assay reagent was added to each well, and the plate was incubated for 2 h at 37°C. After incubation, absorbance was taken at 490 nm. Lymphoma cells treated with the vehicle alone were used as controls. The percent cell viability or cell death induced by GA-DM was calculated using the equation:

$$\left[\frac{Absorbance_{control}-Absorbance_{treated}}{Absorbance_{control}}\right]\;\times \;100$$

All experiments were repeated at least three times, and the data were expressed as percent (%) cell death ± standard error (SE) of triplicate wells.

### Western blot analysis

DB and Toledo lymphoma cells were cultured for 24 h in the presence of vehicle alone or GA-DM (30 μM), as indicated above. Following treatment, cells were washed, and cell lysate was obtained using a standard lysis buffer (10 mM Trizma base, 150 mM NaCl, 1% Triton-X 100) [37, 38]. Equal protein concentrations from DB cell lysates were separated on a 4–12% Bis/Tris NuPage gel (Cat #NP0321BOX Invitrogen, Grand Island, NY). Proteins were transferred onto a nitrocellulose membrane (Pierce, Rockford, IL) and probed with a specific antibody for the detection of caspase-3 (Cat #31A1067, Alexis Biochemicals, Plymouth Meeting, PA). For western blotting, the secondary antibodies used were horseradish peroxidase (HRP) conjugated anti-mouse, anti-rabbit, or anti-goat IgG (Cat #sc-2004, sc-2005, sc-2357, Santa Cruz Biotechnology, Dallas, TX). A monoclonal antibody against β-actin (1:1000, sc-81178, Santa Cruz Biotechnology, Dallas, TX) was used for the protein loading control. Relative protein expression was assessed using ImageJ software (National Institutes of Health, Bethesda, MD) and expressed as relative density ± SEM for each sample [39–41].

### Caspase activation and inhibition assay

Lymphoma cells were treated with different concentrations of GA-DM and subjected to a subsequent assay for cellular activity of caspase-3 [37, 38]. Briefly, 5 × 10^4^ cells were plated in 100 μL of total volume in a 96-well plate and treated with 30 μM of GA-DM for 24 h at 37°C. The plate was then equilibrated for 30 min at room temperature, and 100 μL of Caspase-Glo^®^ 3/7 reagents (Cat #G8090 Promega Corporation, Madison, WI) was added to each well. Luminescence was recorded 30 min after adding reagents using a Floustar Optima microplate reader (BMG, Durham, NC) [37, 38]. To determine caspase activity, cells were treated with GA-DM (30 μM) in the presence or absence of the pan-caspase inhibitor Z-VAD-FMK at a 50 μM final concentration (R&D Systems #FMK001, Minneapolis, MN) [38]. Cells treated with the vehicle alone were used as controls as described. Cells were then incubated at 37°C for 24 h, and cell viability was measured using the MTS assay [37].

### Immunofluorescent staining

DB and Toledo lymphoma cells were cultured in 4-well culture slides (Cat #354104 Corning, Manassas, VA) for 24 h in the presence of vehicle alone or vehicle + GA-DM (30 μM) at 37°C as described above. Following treatment, cells were centrifuged at 1,200 rpm for 5 min to deposit a monolayer of cells from the cell suspension onto the slide. They were then fixed onto the slide with ice-cold 100% methanol, washed with phosphate-buffered saline (PBS) (Cat #P3813 pH 7.4 Sigma-Aldrich, St. Louis, MO) + 0.1% Triton-X 100 (PBST), and blocked with 8% normal horse serum in PBST for 1hr at room temperature. The cells were then incubated with 1:100 HLA-DR L243 mouse monoclonal IgG (Cat #sc-18875 Santa Cruz Biotechnology, Dallas, TX) primary antibody at 4°C for overnight. The next day after a 3 × wash with PBS, the cells were incubated in 1:200 horse anti-mouse IgG DyLight 594 (Cat #DI-2594 Vector Laboratories, Newark, CA) secondary antibody in 5% horse serum for 1hr at room temperature. Nuclear staining was obtained using Vectashield Antifade Mounting Medium with DAPI (Cat #H-1800–10 Vector Laboratories, Newark, CA). After staining, cells were imaged using the Olympus-IX73 microscope.

### Flow cytometry

DB and Toledo cells were cultured for 24 h in the presence of vehicle alone or vehicle + 30 μM of GA-DM at 37°C in a 5% CO_2_ incubator [31, 32]. After treatment, cells were washed twice with PBS and staining buffer (PBS + 1% heat-inactivated BGS) (HyClone) and resuspended in a binding buffer [Cat #556454, BD Biosciences: 0.1 M HEPES (pH 7.4), 1.4 M NaCl, and 25 mM CaCl_2_]. Cells were stained with the HLA-DR4-specific mAb, 359-F10, followed by appropriate secondary antibody labeled with FITC as described previously [42]. Background fluorescence was determined using an irrelevant isotype-matched mAb IN-1 as described [34].

### Peptides and antibodies

Human IgGκ_188–203_ (KHKVYACEVTHQGLSS) peptide was prepared using Fmoc technology and an Applied Biosystems Synthesizer as described previously [32, 42]. Peptide purity (> 99%) and sequence were determined by reverse phase HPLC purification and mass spectroscopy [34]. The IgGκ_188–203_ peptide was dissolved in 1% DMSO (Cat #D2650 Sigma, Burlington, MA) and stored at −20°C until used. The primary antibodies used were human caspase-3 (Cat #31A1067, Alexis Biochemicals, Plymouth Meeting, PA) and β-actin (Cat #sc-81178, Santa Cruz Biotechnology, Dallas, TX). The secondary antibodies used were HRP conjugated anti-mouse, anti-rabbit or anti-goat IgG (Cat #sc-2004, sc-2005, sc-2357, Santa Cruz Biotechnology, Dallas, TX), as described above.

### Antigen presentation assay

Cells (2 × 10^4^ cells/well) were treated with either vehicle alone or 30 μM of GA-DM for 24 h at 37°C in 5% CO_2_ incubator in a 96-well plate. To examine changes in cellular ability of presentation of antigens after treatment, the IgGk_188–203_ peptide (20 μM) was added to the appropriate 96-wells for the last 4 h of incubation. Cells were then washed twice and co-cultured with the IgGk_188–203_ peptide-specific T cell hybridoma (2.18a) for 24 h at 37°C [42]. The plates were spun down, and the production of IL-2 was tested by enzyme-linked immunosorbent assay (ELISA) and expressed as pg/mL ± SEM [31, 32]. The amount of IL-2 in the co-culture supernatant corresponds to activation of CD4^+^ T cells and immune activation/recognition [43]. All assays were repeated at least three times.

### ELISA

To analyze IL-2 cell supernatants from triplicate wells, 96-well plates were evaluated by ELISA (R&D Systems, Minneapolis, MN, USA) according to the manufacturer’s instructions [43]. Anti-IL-2 was purchased from Sigma Aldrich (St Louis, MO, USA; Cat #WH0003558M3–100UG). All the assays were repeated at least three times.

### Statistical analysis

Data from each experimental group were subjected to statistical analysis [31, 36]. ANOVA with post-hoc tests and the student’s *t*-tests were used, as appropriate, in the experiments. Two-sided tests were used in all cases, and *p*-values < 0.05 were considered statistically significant.

## Results

### Triterpenoid GA-DM induces caspase-dependent apoptosis in human lymphoma cells

**Figure 1.**
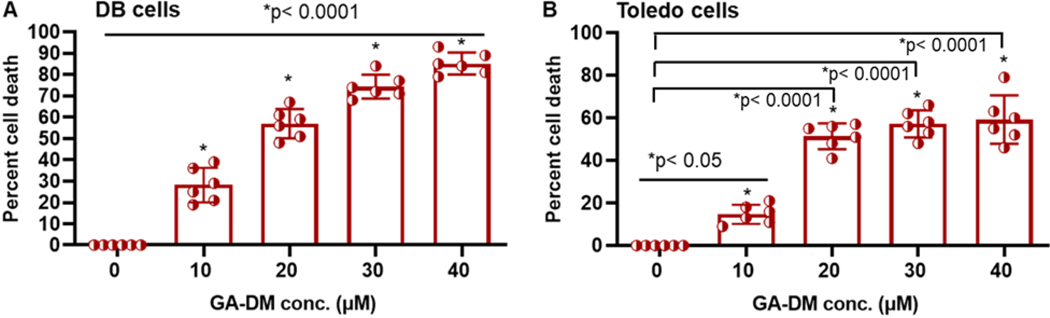
Ganoderic acid DM (GA-DM) treatment reduces cell viability in human lymphoma cells. Human lymphoma cell lines DB (**A**) and Toledo (**B**) were treated with vehicle (DMSO, < 0.01%) alone or GA-DM (10–40 μM) for 24 h at 37°C, followed by the MTS viability assay as described in the Materials and methods. Control cells treated with vehicle alone were utilized to calculate the percent cell death induced by GA-DM, as indicated in the methods. The data shown are results of at least three separate experiments that were performed in triplicate wells. Error bars represent mean ± SD, and the *p*-values compare the treatments to the control. *p* < 0.05 is considered statistically significant.

Human lymphoma cells DB and Toledo were treated with various concentrations of GA-DM (0–40 μM) for 24 h, followed by the MTS cell viability assay (Figure 1). GA-DM treatment of lymphoma cells induced cell death as calculated by the formula described in the methods. A dose-dependent reduction of cell viability was also detected when increased concentrations of GA-DM were used in the cell viability assay. It was noted that 20–40 μM of GA-DM induced more than 50% of DB and Toledo cell death after 24 h of treatment, although repeated experiments showed 30–40 μM of GA-DM consistently induced more than 60% cell death in DB cells (Figure 1, A and B). Interestingly, the cell death percentage of DB and Toledo lines varied depending on GA-DM concentration. DB cells showed increased sensitivity when the dose was increased, but Toledo cells reached a plateau with GA-DM concentrations of 20–40 μM. Overall, these data suggest that GA-DM treatment induces lymphoma cell death with variable sensitivity, and it may be dependent on cell lineage.

**Figure 2.**
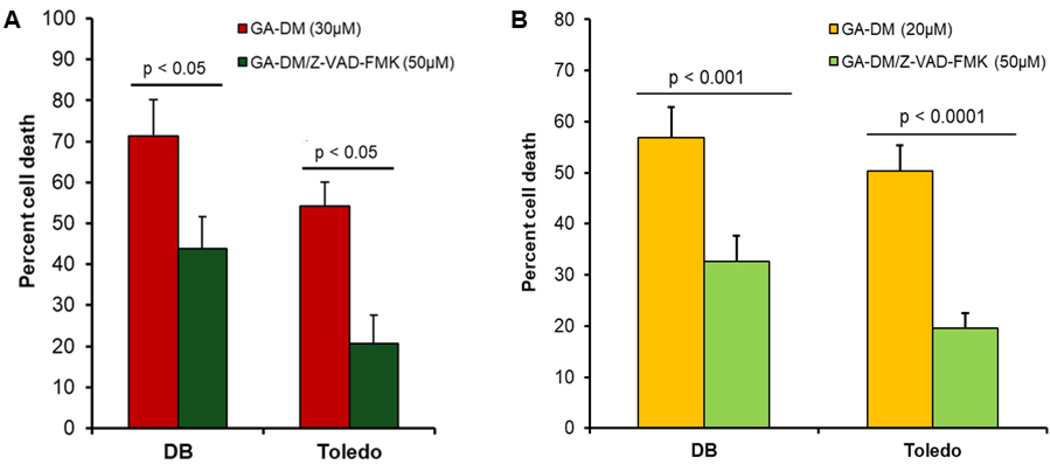
Ganoderic acid DM (GA-DM) treatment induces caspase-3 dependent cell death in human B-cell lymphoma. DB and Toledo B-lymphoma cell lines were treated with GA-DM (30 μM) or vehicle alone and incubated with or without Z-VAD-FMK for 24 h at 37°C, followed by the MTS viability assay and calculation of cell death as described in the methods. (**B**) DB and Toledo B-lymphoma cell lines were also treated with GA-DM (20 μM) or vehicle alone and incubated with or without Z-VAD-FMK for 24 h at 37°C, followed by the MTS viability assay and calculation of cell death. Data are presented as mean ± SD from at least three independent experiments. Significant differences were calculated by Student’s *t-*test, where *p* < 0.05 is statistically significant. Data suggest inhibition of caspases by Z-VAD-FMK blocked GA-DM-induced cell death in lymphoma cells.

To study the mechanisms of GA-DM-mediated cell death in human lymphoma cells, DB cells were treated with GA-DM (30 μM) in the presence or absence of a pan-caspase inhibitor Z-VAD-FMK, which is known to bind to the catalytic site of caspases. Treatment with Z-VAD-FMK significantly blocked GA-DM-induced cell death in both DB and Toledo lymphoma cells (Figure 2A), suggesting that the cell death is caspase dependent. To further study the effects of 20 μM GA-DM on caspase-mediated cell death, DB and Toledo cells were treated with GA-DM (20 μM) in the presence or absence of a pan-caspase inhibitor Z-VAD-FMK. Treatment with Z-VAD-FMK significantly blocked 20 μM of GA-DM-induced cell death in both DB and Toledo cells (Figure 2B), suggesting that 20 μM of GA-DM consistently induces caspase-dependent cell death.

### Triterpenoid GA-DM activates both apoptotic and autophagy markers in human DB lymphoma cells

**Figure 3.**
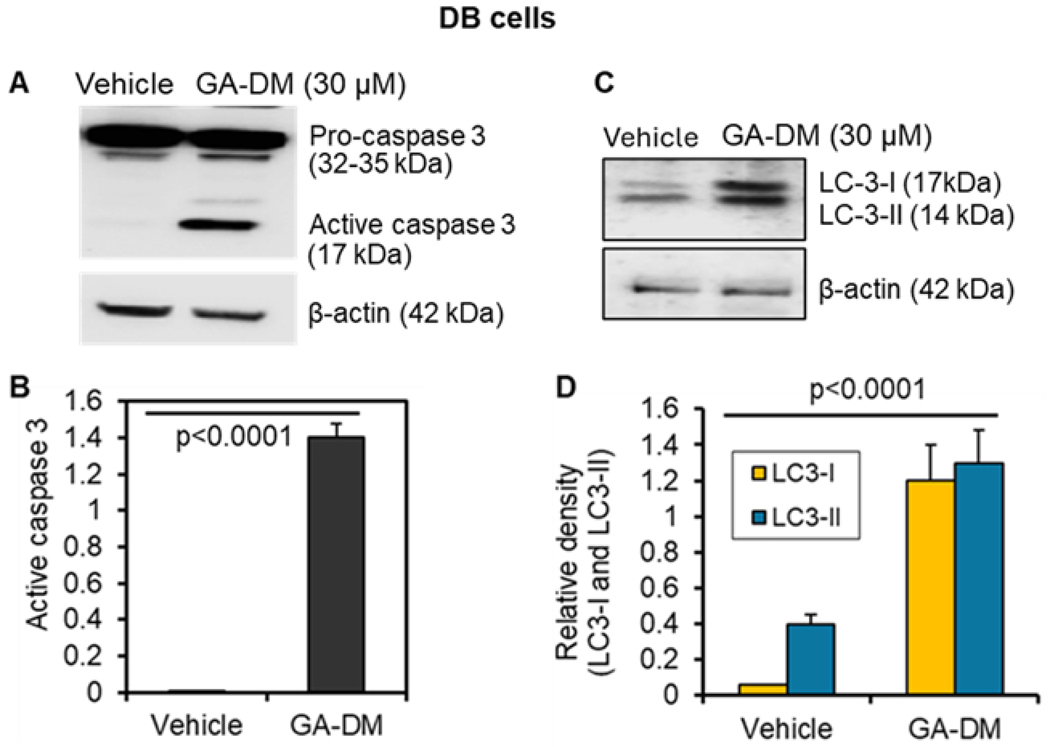
Ganoderic acid DM (GA-DM) treatment activates caspase processing in human DB B-cell lymphoma. (**A**) Western blot analysis showing caspase-3 protein expression and cleavage of active caspase-3 in DB lymphoma cells treated with GA-DM (30 μM) or vehicle alone (control) as described in the methods. β-actin was used as a loading control. (**B**) Quantitative analysis of active caspase-3 protein band by ImageJ software. (**C**) Western blot analysis also shows that GA-DM treatment upregulates autophagic protein LC3 in DB cells. (**D**) Quantitative analysis of LC3 protein bands by ImageJ software. The data are representative of three separate experiments. Significant differences were calculated by Student’s *t-*test, and *p* < 0.05 is statistically significant.

DB cells were then subjected to western blotting to detect active caspase-3. Western blot analysis showed that processed caspase (active caspase-3) was only detected in GA-DM (30 μM) treated DB cells and not in the control group (Figure 3A). Quantitative analysis of protein bands showed a drastic increase in active caspase-3 in GA-DM-treated DB cells (Figure 3B), suggesting that GA-DM induces apoptosis in lymphoma cells. Western blot analysis showed enhanced LC3-I to LC3-II conversion in GA-DM-treated DB cells (Figure 3C), indicating the induction of autophagy. Quantitative analysis of protein bands by ImageJ showed a significant increase in both LC3-I and LC3-II proteins in GA-DM-treated DB cells as compared to controls (Figure 3D). GA-DM-treated Toledo cells also showed similar expression patterns of LC3 and caspase-3 proteins as found by western blot analysis (Figure S1). This data indicates that GA-DM treatment may induce both autophagy and apoptosis in human DB and Toledo lymphoma cells.

### GA-DM treatment enhances HLA class II-restricted antigen presentation by human lymphoma cells

**Figure 4.**
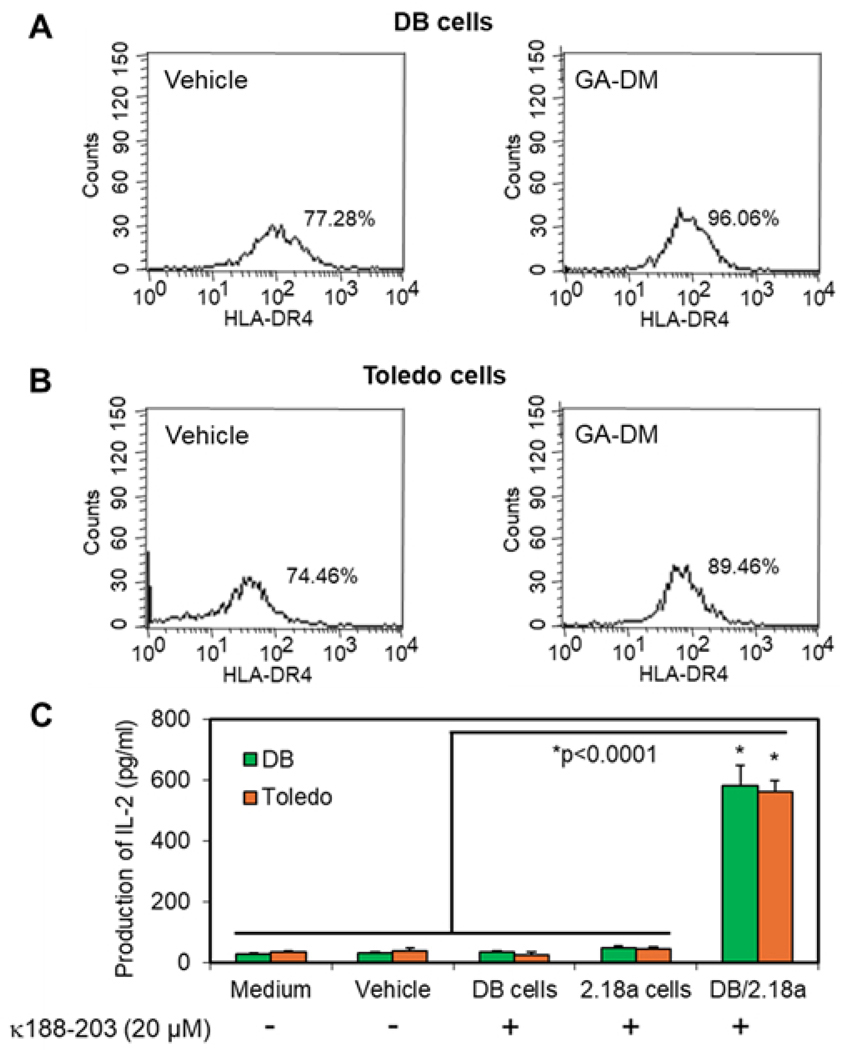
HLA class II DR4 expression in DB and Toledo lymphoma cells and CD4^+^ T cell recognition of B-cell lymphoma. (**A**–**B**) DB and Toledo lymphoma cells were transduced with HLA-DR4, treated with vehicle alone or GA-DM (30 μM), and analyzed by flow cytometric analysis for cell surface expression of HLA-DR4 proteins. (**C**) Antigen presentation by B-cell lymphoma cells to CD4^+^ T cells. DB and Toledo lymphoma cells were incubated with IgGκ_188–203_ peptide for overnight, washed, and co-cultured with the peptide-specific T cell hybridoma (2.18a) for 24 h. The production of IL-2 was quantitated by ELISA and expressed as pg/mL ± SD of triplicate wells of at least three independent experiments.

To investigate whether GA-DM treatment influences HLA class II protein expression on the cell surface, flow cytometric analysis was performed. First, DB and Toledo cells were transduced with HLA-DR4 (DRB1*0401), and the expression of DR4 was confirmed by flow cytometry. HLA-DR4 protein expression was markedly increased (77.28% vs. 96.06% in DB cells and 74.46% vs. 89.46% in Toledo cells) in both DB and Toledo cells following treatment with 30 μM of GA-DM (Figure 4, A and B). We then tested whether DB and Toledo lymphoma cells present antigens to CD4^+^ T cells. An antigen presentation assay was performed, which showed that both DB and Toledo cells can present the IgGk_188–203_ peptide to CD4^+^ T cells in the context of HLA-DR4 (Figure 4C).

**Figure 5.**
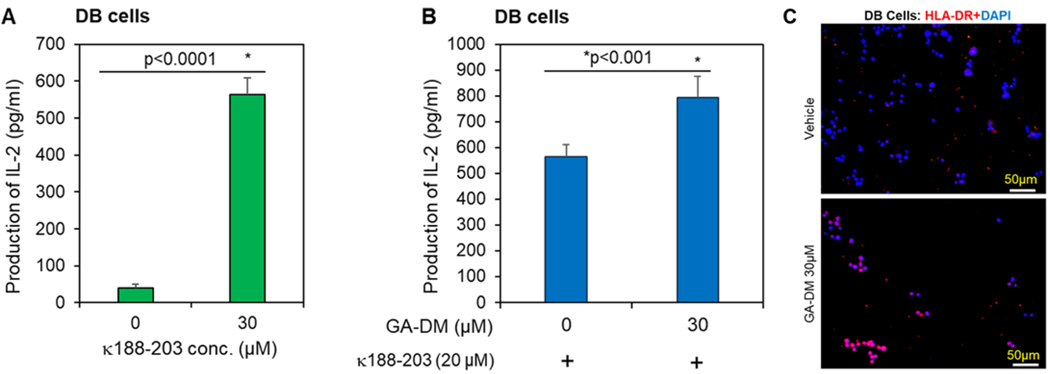
Ganoderic acid DM (GA-DM) treatment enhances HLA class II-restricted antigen presentation by B-cell lymphoma. (**A**) IgGκ_188–203_ peptide presentation and immune recognition of HLA-DR4 expressing DB cells. Cells were incubated with the IgGκ_188–203_ peptide for overnight, washed, and cocultured with the IgGκ_188–203_ peptide-specific T cell hybridoma for 24h. (**B**) Cells were treated with GA-DM (30 μM) or vehicle alone for overnight, followed by incubation with IgGκ_188–203_ peptide for another 4 h, washed, and co-cultured with the peptide-specific T cell hybridoma (2.18a) for 24 h. T cell production of IL-2 was measured by ELISA and expressed as pg/mL ± SD of triplicate wells of at least three independent experiments. Effects of GA-DM on Ag presentation were calculated as compared to vehicle controls. (**C**) DB cells treated with vehicle alone or GA-DM (30 μM) overnight were also stained with HLA-DR (L243) and anti-mouse IgG DyLight 594, as described in the methods. Nuclear staining was obtained using DAPI. Representative images were taken under a fluorescence microscope at 20× magnification. Bar = 50 μm.

We also examined whether GA-DM treatment affects functional antigen presentation to CD4^+^ T cells. DB lymphoma cells were treated with vehicle alone or GA-DM (30 μM) and tested for antigen presentation to CD4^+^ T cells (Figure 5, A and B). The level of immune recognition was measured by the amount of IL-2 production in the supernatants collected after co-incubation of treated or control cells with the IgGk_188–203_ peptide-specific CD4^+^ T cells. Data showed that GA-DM treatment enhanced immune recognition of DB lymphoma cells via the HLA class II pathway. The immune response induced by GA-DM treatment may be biologically significant since smaller numbers of viable cells were still able to enhance antigen presentation and CD4^+^ T cell activation. Cells were also stained with HLA-DR, where brighter/intense staining was observed in the GA-DM (30 μM) treated group compared to the control (Figure 5C). Similarly, Toledo cells treated with GA-DM also showed increased immune recognition via the HLA class II pathway (Figure S2). Taken together, these data suggest that GA-DM has the potential to increase HLA class II expression and enhance immune recognition of B-cell lymphomas via the HLA class II pathway. Additionally, it may represent a valuable tool for tumor clearance.

### Crosstalk between autophagy and apoptosis may enhance CD4^+^ T cell recognition of lymphoma cells

**Figure 6.**
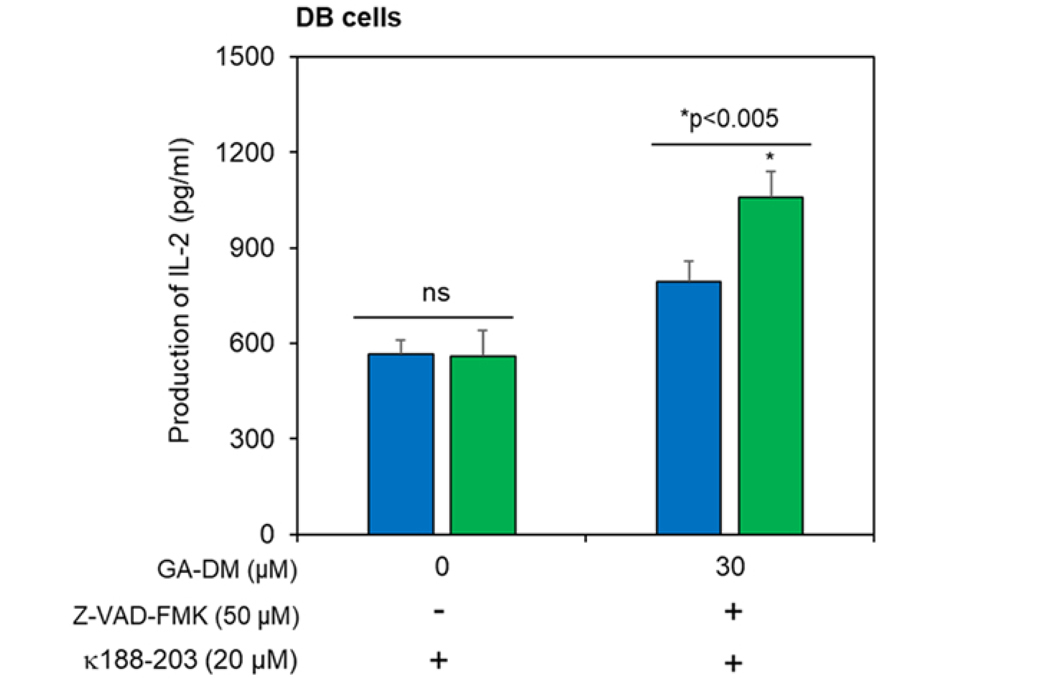
Inhibition of caspases enhances CD4^+^ T cell recognition of lymphoma cells. HLA-DR4-expressing DB cells were treated with GA-DM and/or Z-VAD-FMK as described in the methods and shown in the figure. The IgGκ_188–203_ peptide was added for the last 4 h. Cells were then washed and cocultured with the peptide-specific T cell hybridoma line (2.18a) for 24 h. Production of IL-2 in the cell supernatants was quantitated by ELISA. Experiments were repeated at least three times, and data were expressed as mean ± SD of triplicate wells. Significant differences were calculated by Student’s *t-*test, where *p* < 0.05 is statistically significant.

We have recently shown that GA-DM induces mitochondrial dysfunction, presumably by depolarizing mitochondrial membrane potential, activating the downstream caspase cascades, and inducing apoptotic cell death in lymphoma [44]. In the current study, we tested whether blocking caspase processing by Z-VAD-FMK influences GA-DM-mediated antigen presentation to CD4^+^ T cells (Figure 6). DB cells were treated with GA-DM in the presence or absence of Z-VAD-FMK (50 μM) and IgGk_188–203_ peptide, followed by the addition of peptide-specific T cell hybridoma. Analysis of IL-2 in the culture supernatant showed that blocking active caspases by Z-VAD-FMK enhanced GA-DM-mediated antigen presentation to CD4^+^ T cells. Although GA-DM-induced caspase caused apoptosis, it also enhanced HLA class II antigen presentation, indicating that the remaining viable tumor cells were capable of inducing T-cell responses.

**Figure 7.**
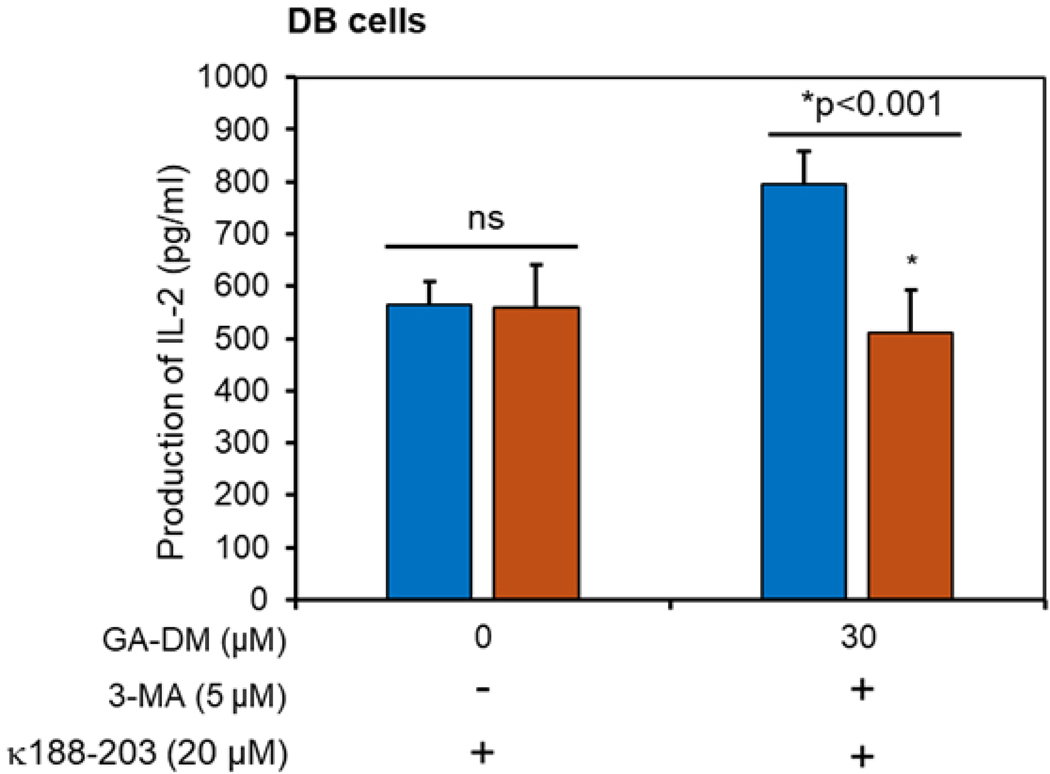
Blocking autophagy decreased CD4^+^ T cell recognition of lymphoma cells. HLA-DR4 expressing DB cells were treated with 3-MA and/or GA-DM for as described in the methods and shown in the figure. The IgGκ_188–203_ peptide was added for the last 4 h. Cells were washed and cocultured with the peptide-specific T cell hybridoma 2.181 for 24 h. Production of IL-2 in the culture supernatants was quantitated by ELISA. Experiments were repeated at least three times, and data were expressed as mean ± SD of triplicate wells. Significant differences were calculated by Student’s *t*-test, where *p* < 0.05 is statistically significant.

In addition to apoptosis, GA-DM treatment also induced autophagy by upregulating LC3, as shown in Figure 3C, indicating GA-DM may induce crosstalk between autophagy and apoptosis in lymphoma cells. Enhanced autophagy may promote antigen presentation via the HLA class II pathway, and it is critical for the development of immunity against malignant tumors. To examine whether GA-DM-induced autophagy enhances T cell activation, an antigen presentation assay was performed in DB cells in the presence or absence of a known autophagy inhibitor (3-MA, 5 μM). Interestingly, enhanced antigen presentation by GA-DM was blocked by 3-MA (Figure 7), suggesting that GA-DM-induced autophagy and enhanced immune recognition can be exploited for immunotherapy of lymphoma. These results are consistent with the pan-caspase inhibitor Z-VAD-FMK treatment and antigen presentation data, suggesting GA-DM-induced crosstalk between autophagy and apoptosis may increase antigen presentation and CD4^+^ T cell recognition of lymphoma cells.

## Discussion

Cancer remains one of the leading causes of mortality worldwide, with conventional therapies such as chemotherapy and radiation therapy often limited by toxicity, resistance, and incomplete eradication of malignant cells [45–47]. In recent years, immunotherapy has emerged as a transformative approach, harnessing the immune system to target and eliminate malignant cells. However, the efficacy of immunotherapy is often hindered by the immunosuppressive tumor microenvironment (TME) and the inability of tumor cells to optimally present antigens to T cells [48–50]. These challenges underline the need for novel therapeutic strategies that can simultaneously induce cancer cell death and enhance immune recognition. The interplay between autophagy and apoptosis is a complex and context-dependent process that plays a central role in cellular decision-making [22, 51]. Understanding this interplay is crucial for developing targeted therapies for lymphoma as well as other inflammatory diseases.

The immune response to autophagy is a complex process, as autophagy influences both innate and adaptive immunity [52, 53]. Autophagy not only helps maintain cellular homeostasis by clearing damaged organelles and cellular proteins, but also actively participates in immune regulation. The immune system often relies on antigen presentation to recognize and target tumor cells for elimination [54, 55]. Enhancing this process through chemoimmunotherapy has become a major focus in recent cancer research, offering hope for developing more effective and targeted therapies.

The Toledo and DB cell lines are derived from human DLBCL, a highly aggressive and common form of NHL. The DB cell line originates from the ascites of a 45-year-old male patient and carries the EZH2 Y641N mutation, classifying it as a germinal center B-cell-like (GCB) subtype. Treatment with GA-DM led to a dose-dependent reduction in cell viability in both cell lines. However, DB cells were more sensitive to GA-DM, with over 60% cell death at 30–40 μM, while Toledo cells showed a less pronounced response, plateauing within the same concentration range. These results suggest GA-DM is effective in inducing cell death in DLBCL cells, particularly in the DB cell line.

Dysregulation of cellular caspase-3 activity can contribute to tumor development by allowing cells to escape cell death by apoptosis [56, 57]. Caspase-3 is a crucial player in the execution phase of apoptosis induction, mediating the dismantling of the cell in a controlled manner. Its activation and regulation are thought to be tightly controlled to ensure that apoptosis occurs only when necessary, maintaining cellular and tissue homeostasis, health, and disease. Our study suggests that GA-DM treatment activates caspase-3, inducing lymphoma cell death. On the other hand, GA-DM also induced LC3-II in lymphoma cells. LC3 is an important player in the induction of autophagy, which is vital for regulating immune recognition [51, 58, 59]. It contributes to antigen presentation, maintenance of immune homeostasis, and inflammation control, while its dysregulation is linked to disruption of immune homeostasis and induction of various diseases. Understanding the role of this microtubule-associated protein, LC3, in immunity provides insights into developing better therapeutic strategies for malignant and inflammatory disorders.

Chemotherapy can disrupt cellular functions in cancer cells, leading to apoptosis [22, 57, 60]. But induction of autophagy by targeted chemotherapy might have a dual role in inducing cellular stress, disrupting cell function, and contributing to tumor cell death [19, 61]. So, the interaction between autophagy and chemotherapy-induced apoptotic cell death could be complex, which requires further investigation. Here, GA-DM-induced autophagy played a pro-death role in killing B-lymphoma cells. Thus, modulating autophagy by GA-DM could influence cellular responses and improve treatment outcomes. The interplay between autophagy and chemotherapy-induced cell death can be better regulated by treating cells with GA-DM, which is complex and critical in cancer treatment [19, 61, 62]. As mentioned above, autophagy is a double-edged sword in chemotherapy as well as immunotherapy. While it can determine cellular fate by protecting cancer cells and inducing resistance, it can also contribute to cell death in specific scenarios like GA-DM treatment. Our data suggests that GA-DM treatment could influence autophagy pathways to tip the balance toward killing B-lymphoma cells. Thus, the natural product GA-DM could be combined with existing chemotherapeutics to modulate the interplay between autophagy and apoptosis to clear malignant lymphoma cells.

Apoptosis is long thought to be a preferred way to kill malignant cells by chemotherapy as it induces programmed cell death without inflammation [13, 59, 63]. However, malignant cells often develop resistance to cell death by apoptosis, making it a major focus of careful drug design and selection. In some cases, autophagy induced by chemotherapy may support apoptosis by providing energy, although in other cases, chemotherapy may hinder apoptosis [13, 21, 60]. Recent strategies focus on resensitizing tumor cells to apoptosis by targeting anti-apoptotic proteins or using combination approaches. Thus, understanding the crosstalk between apoptosis and autophagy by natural triterpenoids is critical for improving patient outcomes. HLA class II molecules are often involved in presenting antigens to CD4^+^ T cells [34, 35]. They can present both exogenous and endogenous antigens and induce antitumor immunity by supporting the function of CD8^+^ T cells. When cells undergo apoptosis, they’re engulfed by APCs like dendritic cells and macrophages, processed and presented via the HLA class II pathway. The induction of enhanced autophagy by triterpenoid GA-DM may favor cross-presentation where HLA class I can present tumor antigens to activate CD8^+^ T cells, clearing B-lymphoma cells.

Antigen presentation, mediated by major histocompatibility complex (MHC) class I and II molecules, is a fundamental principle of adaptive immunity [54, 64]. Tumor cells often downregulate cell surface MHC protein expression to evade immune detection, limiting the efficacy of therapy such as immune checkpoint blockers and cancer vaccines. Thus, strategies to enhance antigen presentation, especially in combination with apoptotic agents, may hold promise for overcoming immune escape and improving therapeutic outcomes. However, the development of anticancer agents that can concurrently induce autophagy, apoptosis, and enhance antigen presentation and immune recognition remains an unmet need in cancer chemoimmunotherapy. In this study, we report the development and characterization of a natural plant derivative that uniquely integrates these three mechanisms: induction of autophagy, promotion of apoptosis, and enhancement of antigen presentation. By simultaneously inducing autophagy, triggering tumor cell death, and augmenting immune recognition, this drug represents a promising approach to overcoming the limitations of existing cancer therapies. Our findings not only provide insights into the crosstalk between autophagy, apoptosis, and antigen presentation but also pave the way for a new class of immunotherapeutic agents with broad applications in cancer chemoimmunotherapy.

The limitations of the study are as follows: While our study provides important insights into the interplay between autophagy, apoptosis, and antigen presentation in B-lymphoma cells treated with GADM, several limitations must be acknowledged. First, the mechanistic conclusions are largely based on in vitro findings, which may not fully capture the complexity of the TME or immune system interactions in vivo. Second, although our data suggests that GA-DM induces both autophagy and apoptosis, the precise molecular pathways governing their crosstalk remain to be fully elucidated. Given that autophagy can have both pro-survival and pro-death roles depending on the context, a deeper understanding of the regulatory checkpoints is necessary to harness its therapeutic benefits effectively. While enhanced antigen presentation was observed, we did not directly assess T cell activation or cytotoxic responses in vivo, which are critical for establishing the immunogenic potential of GA-DM treatment. The variability in MHC expression levels among different tumor types may also limit the generalizability of these findings in other tumors. Finally, the dual role of autophagy in promoting immune evasion or activation highlights the need for careful therapeutic modulation, as indiscriminate activation may yield unintended effects on immune recognition of B-cell lymphomas. Future studies should focus on in vivo validation, exploration of combinatorial strategies with checkpoint inhibitors or other chemotherapeutics, and a comprehensive characterization of immune cell dynamics to fully realize the clinical potential of GA-DM in cancer chemoimmunotherapy.

## Supplementary Material

1003216_sup_1 Blumenstock et al

The supplementary figures for this article are available at: https://www.explorationpub.com/uploads/Article/file/1003216_sup_1.pdf.

## Data Availability

The data used to support the findings of this manuscript are available from the corresponding authors upon reasonable written request after the publication.

## Abbreviations

APCs: antigen-presenting cells
ATCC: American Type Culture Collection
DLBCL: diffuse large B-cell lymphoma
DMSO: dimethyl sulfoxide
ELISA: enzyme-linked immunosorbent assay
FBS: fetal bovine serum
GA-DM: ganoderic acid DM
MHC: major histocompatibility complex
NHL: non-Hodgkin’s lymphoma
TME: tumor microenvironment
