# Supplementary material for: Crosstalk between autophagy and apoptosis in initiating antitumor immune responses in human lymphoma cells
Source: Explor Immunol. Author manuscript; Available in PMC 2026 Mar 13. (PMC12981232; doi:10.37349/ei.2025.1003216)
Supplement: 1003216_sup_1 Blumenstock et al [file NIHMS2134839-supplement-1003216_sup_1_Blumenstock_et_al.pdf]

Supplementary materials

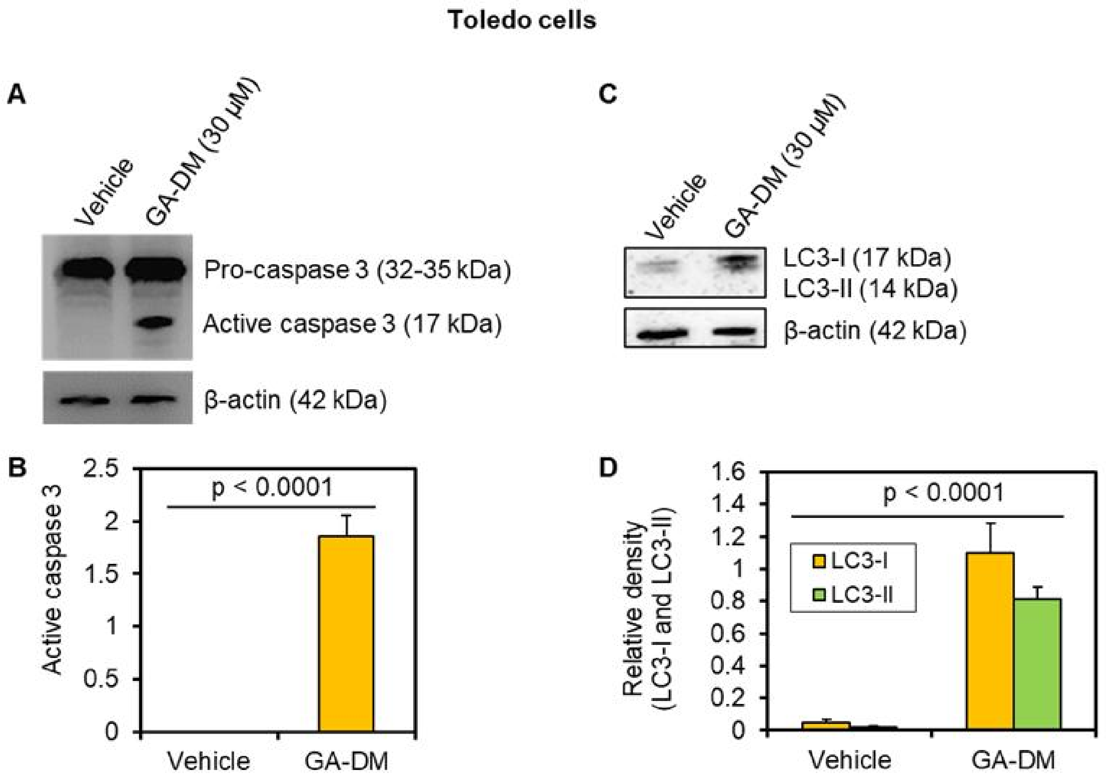

Figure S1. Ganoderic acid DM (GA-DM) treatment activates caspase processing in human Toledo B-cell lymphoma. (**A**) Western blot analysis showing caspase-3 protein expression and cleavage of active caspase 3 in Toledo lymphoma cells treated with GA-DM (30 µM) or vehicle alone (control) as described in the methods. β-actin was used as a loading control. (**B**) Quantitative analysis of active caspase 3 protein band by ImageJ software (**C**) Western blot analysis also shows that GA-DM treatment upregulates autophagic protein LC3 in Toledo cells. (**D**) Quantitative analysis of LC3 protein bands by ImageJ software. The data are representative of three separate experiments. Significant differences were calculated by Student's *t*-test, and $p < 0.05$ is considered statistically significant.

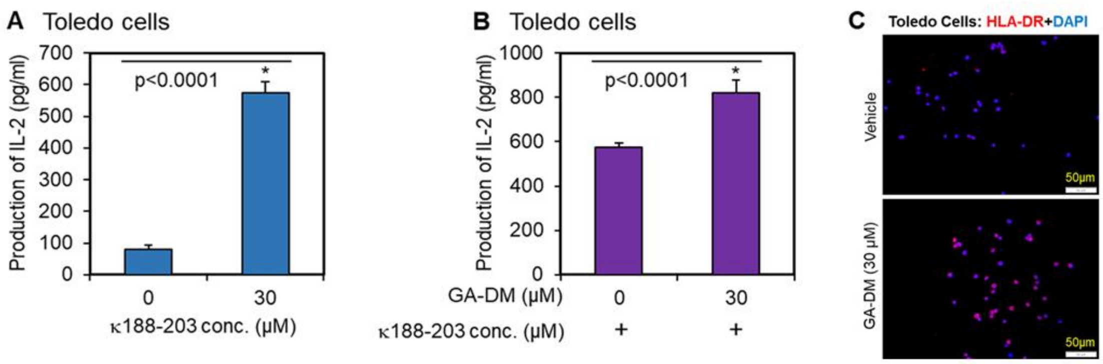

Figure S2. Ganoderic acid DM (GA-DM) treatment enhances HLA class II-restricted antigen presentation in Toledo B-cell lymphoma. (**A**) IgGκ$_{188–203}$ peptide presentation and immune recognition of HLA-DR4 expressing Toledo cells. Cells were incubated with the IgGκ$_{188–203}$ peptide for overnight, washed, and cocultured with the IgGκ$_{188–203}$ peptide-specific T cell hybridoma for 24 h. (**B**) Cells were treated with GA-DM (30 µM) or vehicle alone for overnight, followed by incubation with IgGκ$_{188–203}$ peptide for another 4 h, washed, and co-cultured with the peptide-specific T cell hybridoma (2.18a) for 24 h. T cell production of IL-2 was measured by ELISA and expressed as pg/mL ± SD of triplicate wells of at least three independent experiments. Effects of GA-DM on Ag presentation were calculated as compared to vehicle controls. (**C**) Toledo cells treated with vehicle alone or GA-DM (30 µM) for overnight were also stained with HLA-DR (L243) and anti-mouse IgG DyLight 594, as described in the methods. Nuclear staining was performed using DAPI. Representative images were taken under a fluorescence microscope at 20× magnification. Bar = 50 µm.
